# Genetic diversity and population structure analysis based on the high density SNP markers in Ethiopian durum wheat (*Triticum turgidum* ssp. *durum*)

**DOI:** 10.1186/s12863-020-0825-x

**Published:** 2020-02-12

**Authors:** Admas Alemu, Tileye Feyissa, Tesfaye Letta, Bekele Abeyo

**Affiliations:** 1 Department of Microbial, Cellular and Molecular Biology, Addis Ababa University, P.O.Box 1176, Addis Ababa, Ethiopia; 2 Department of Biology, Debre Tabor University, Debre Tabor, Ethiopia; 3 Oromia Agricultural Research Institute, Addis Ababa, Ethiopia; 4 International Maize and Wheat Improvement Center (CIMMYT), Addis Ababa, Ethiopia

**Keywords:** Ethiopian durum wheat, Genetic diversity, Population structure, SNPs, Landraces, Improved varieties

## Abstract

**Background:**

Ethiopia has been considered as a center of diversity and the second possible center of domestication of durum wheat. Genetic diversity and population structure analysis in the existing Ethiopian durum wheat germplasm have enormous importance in enhancing breeding effort and for sustainable conservation. Hence, 192 Ethiopian durum wheat accessions comprising 167 landraces collected from major wheat-growing areas of the country and 25 improved varieties released from Debre Zeit and Sinana Agricultural Research Centers, Ethiopia in different years (1994–2010) were assembled for the current study.

**Results:**

The panel was genotyped with a High-density 90 K wheat SNP array by Illumina and generated 15,338 polymorphic SNPs that were used to analyze the genetic diversity and to estimate the population structure. Varied values of genetic diversity indices were scored across chromosomes and genomes. Genome-wide mean values of Nei’s gene diversity (0.246) and polymorphism information content (0.203) were recorded signifying the presence of high genetic diversity within this collection. Minor allele frequency of the genome varied with a range of 0.005 to 0.5 scoring a mean value of 0.175. Improved varieties clustered separately to landraces in population structure analysis resulted from STRUCTURE, PCA and neighbor joining tree. Landraces clustering was irrespective of their geographical origin signifying the presence of higher admixture that could arise due to the existence of historical exchanges of seeds through informal seed system involving regional and countrywide farming communities in Ethiopia.

**Conclusions:**

Sustainable utilization and conservation of this rich Ethiopian durum wheat genetic resource is an irreplaceable means to cope up from the recurrent climate changes and biotic stresses happening widely and thereby able to keep meeting the demand of durum productivity for the ever-growing human population.

## Background

Modern wheat cultivars generally refer to two species: hexaploid bread wheat, *Triticum aestivum* (2n = 6x = 42, AABBDD), and tetraploid/hard or durum wheat, *T. durum* (2n = 4x = 28, AABB) used for pasta, macaroni, couscous and low-rising bread, and the former accounts for about 95% of world wheat production and durum covers the other 5% [1].

In Ethiopia, wheat is the second most widely produced cereal crop after corn and the third most important staple food behind corn and sorghum [2]. Hard red wheat accounts for about 75–80% of the national production, while durum makes up roughly 10–15% [2]. Wheat has versatile uses in making various foods and drinks, such as bread, ‘Kolo’ (traditional Ethiopian snack made from wheat mixed with barley, chickpea and other legumes and roasted in a clay griddle), ‘Tella’ (traditional Ethiopian beer), pasta, macaroni, biscuit, cake, and others. Additionally, wheat straw is commonly used as a roof thatching material and as animal feed in most wheat-growing rural areas of Ethiopia. Hence, increasing wheat production has been a national goal to decrease the gap between production and human consumption especially in view of the fastest-growing population as compared to production.

Durum wheat is the result of two successful domestication events by ancient farmers: first, from wild emmer (*Triticum turgidum* ssp. *dicoccoides*) to domesticated emmer (*T. turgidum* ssp. *dicoccum*) with the loss of fragility of spikes (disarticulation into spikelets), and second, from cultivated emmer to durum with the appearance of naked kernels or free threshing kernel [3]. Ethiopia is one of the few countries that has been served as the center of primary gene pool for various crops [4–6]. Ethiopian biodiversity institute (EBI), formerly known as Institute of biodiversity conservation (IBC), has maintained more than 60,000 accessions of different crops in its gene bank and of these, 7000 are durum wheat accessions accounting 12% from the total [7, 8]. Besides, up to recent time, agricultural research centers and institutions have been involved in collecting and conserving Ethiopian durum wheat landrace accessions in the country. Due to its uniqueness, Ethiopian durum wheat has been served as a center of focus for genetic studies and the source of novel alleles [9–14]. Vavilov [4] and Zohary [15] reported the presence of high genetic diversity in Ethiopian durum wheat and recent studies specified uniqueness of Ethiopian durum landraces form the Fertile Crescent collections (primary center of domestication) and considered as the possible second center of domestication for the crop [3]. Durum wheat is long established in the country and it was likely introduced into the northern highlands of Ethiopia around 3000 BC [16]. Previous studies indicated the existence of high genetic variation of cultivated durum wheat in Ethiopia that arises due to the wide range of agro-ecological conditions coupled with diverse farmers’ culture [11, 12, 17–22]. Ethiopian farmers have grown durum wheat since immemorial time, mostly under adverse environmental conditions and they developed a broad gene pool of durum wheat landraces adapted to various environmental conditions [23]. In Ethiopia, durum wheat is commonly planted on heavy black clay soils (vertisols) of the highlands between 1800 and 2800 masl [23].

Mechanisms of detecting and analyzing genetic diversity have gradually progressed from Mendelian survey of discrete morphological traits to molecular examinations of DNA variation [24]. Genetic diversity analysis is a critical component of plant genetics, breeding, conservation and evolution [25]. Understanding the existing genetic divergence and distribution of crop species has paramount importance for conservation and selection of parents with diverse genetic backgrounds, thereby rendering crop improvement more efficient [22].

Single nucleotide polymorphisms (SNPs) are the most abundant class of DNA markers. Lower rates of recurrent mutation make them evolutionarily stable. They are excellent markers for studying complex genetic traits and for understanding the genomic evolution. They have been widely used in genome-wide association studies, genetic resource characterization, marker-assisted breeding and genomic selection [26]. Hybridization arrays/microarrays have been used as a preeminent solution to develop SNPs in complex polyploid genomes such as wheat [27]. Once a comprehensive SNP data set is available for a species, a well-designed microarray can be produced; and generally, the technology is then cost-efficient and the process is relatively convenient. The technology avoids the risk of miscalling diversity on homoeologous genomes and its power recently increased 100-fold in wheat moving from 9 K [28] to 820 K [29] genome-wide SNPs. The 90 K wheat SNP array [27] has been successfully used for genetic diversity analysis, genome-wide association mapping and construction of high-density consensus maps in both bread and durum wheat [12, 30–32].

Molecular characterization of Ethiopian durum wheat accessions has been investigated in DNA markers with a very limited number, such as microsatellites [11, 20, 21]. However, except in a single attempt that has made to characterize Ethiopian durum wheat landraces collected by EBI siding with Mediterranean durum wheat [12], the germplasm has not been extensively investigated with a high density SNP markers. Hence, the present study aimed to assess the genetic diversity and population structure of 167 landraces and 25 improved varieties collected and maintained at Debre Zeit and Sinana Agricultural Research Centers, Ethiopia with a 90 K wheat SNP array.

## Results

### SNP markers distribution

**Fig. 1:**
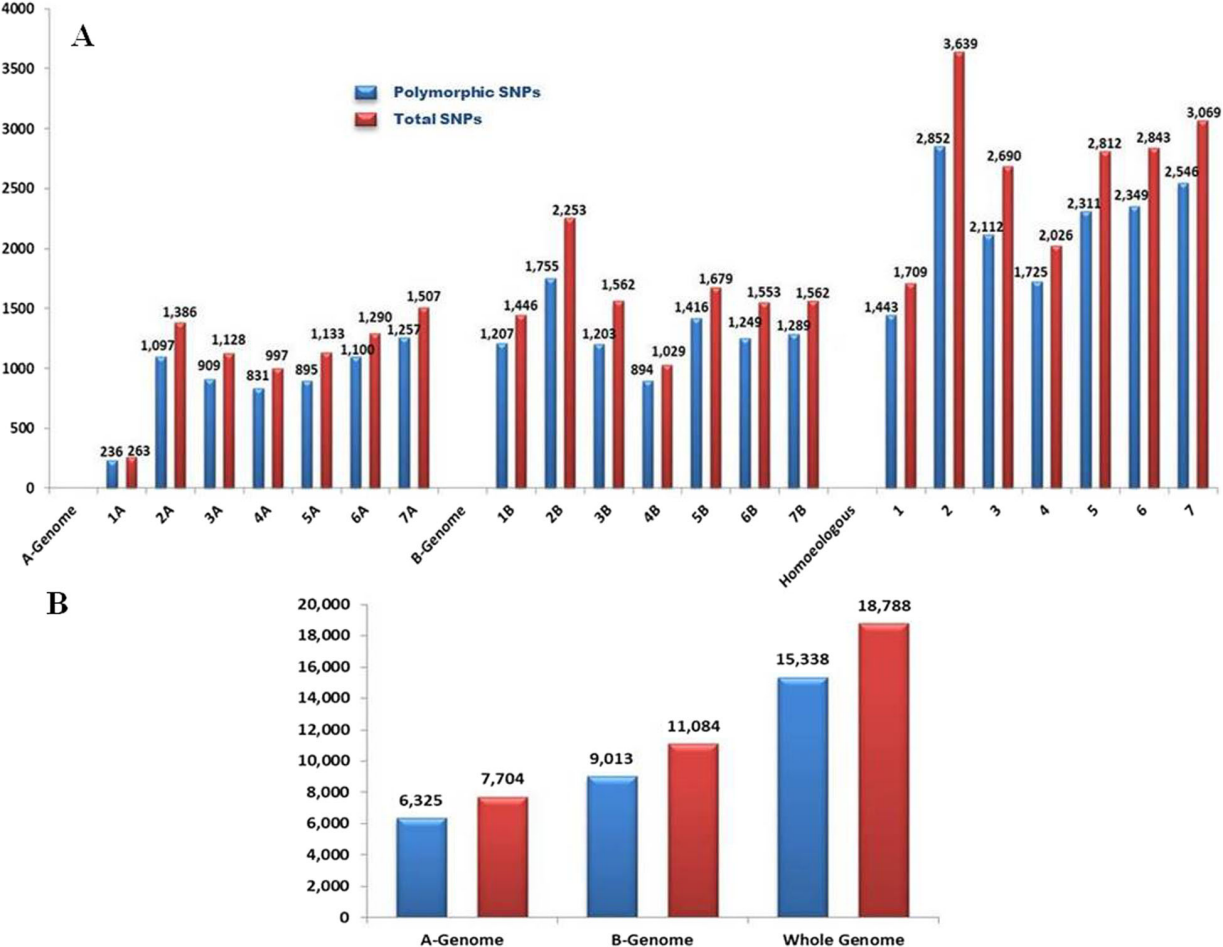
Distribution of SNPs generated from the 90 K Illumina iSelect SNPs array across chromosomes (**A**) and genomes (**B**) in 192 Ethiopian durum wheat accessions

From 81,587 SNP probes available on the chip, 30,510 SNP calls (23,354 polymorphic SNPs) were reproducible in the current Ethiopian durum wheat panel. From these markers, 18,788 SNPs had a known position but only 15,338 (81.63%) were polymorphic and used for the current study (Additional file 2: Table S2). The smallest number of SNP markers were recorded on chromosome 1A (263 SNPs) while the highest on chromosome 2B (2253 SNPs) (Fig. 1-a). Chromosome 2B also contributed the highest number of polymorphic SNP markers (1755 SNPs) while the smallest on chromosome 1A (236 SNPs). Considering the distribution of SNPs across homoeologous chromosomes, group two scored the highest number of SNP markers (3639 SNPs of which 78.38% is polymorphic) while the smallest number on group one with 1709 SNPs of which 84.43% was polymorphic. Higher number polymorphic SNP markers were recorded on B genome (9013 SNPs) than the A genome (6325 SNPs) in Ethiopian durum wheat accessions (Fig. 1-b).

### Genetic diversity analysis

**Fig. 2:**
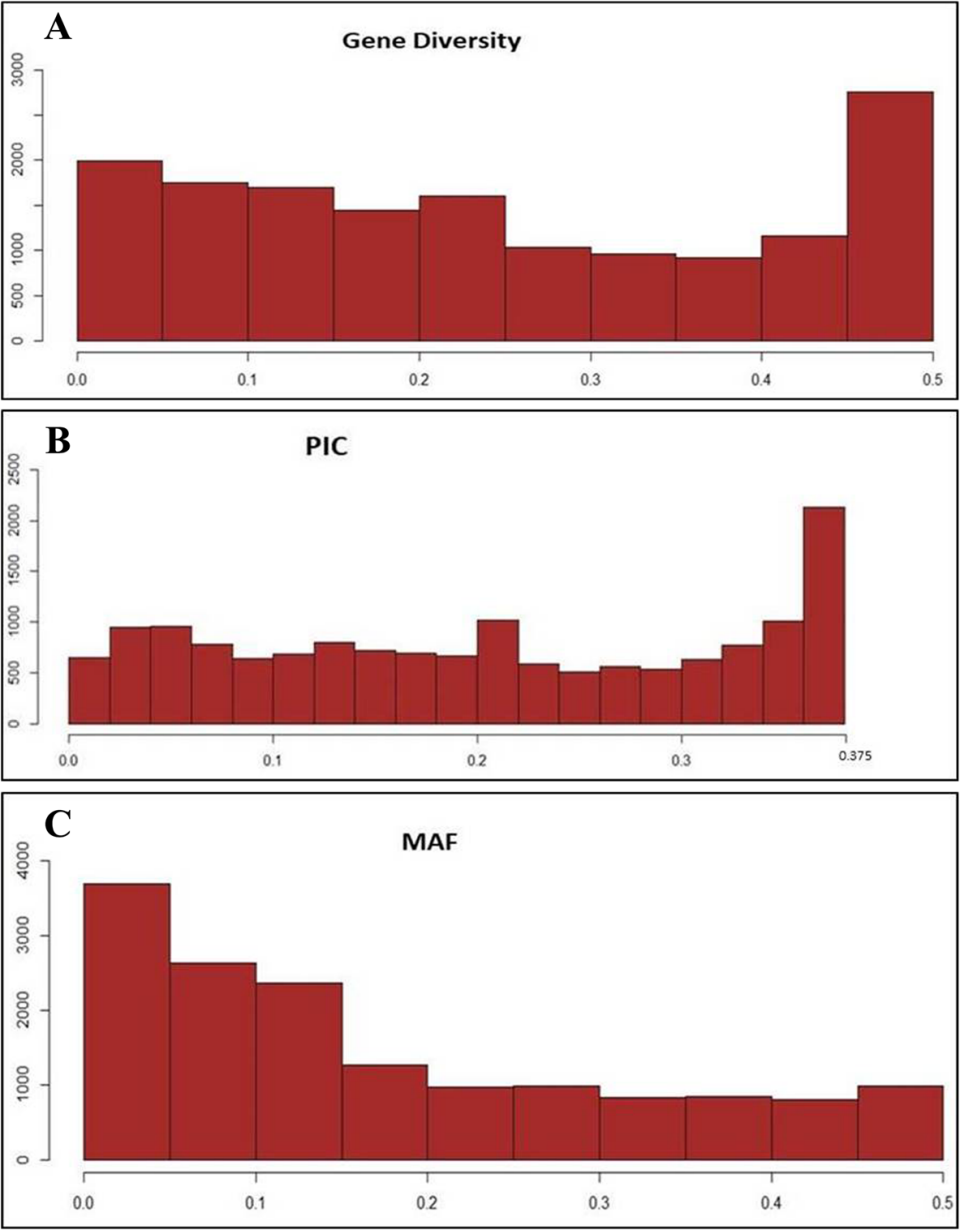
Frequency distribution of Nei’s gene diversity (**A**), polymorphic information content (PIC) (**B**) and minor allelic frequency (MAF) (**C**) of 15,338 polymorphic SNPs generated from Ethiopian durum wheat accessions

The SNP markers exhibited a wide range of polymorphic information content (PIC) and Nei’s gene diversity across chromosomes and genomes of Ethiopian durum wheat. Frequency distribution of SNPs for gene diversity, polymorphic information content, and frequency of the minor allele values of the genome is presented in Fig. 2-a, Fig. 2-b, and Fig. 2-c, respectively. While a detail of the frequency distribution of SNP markers across chromosomes is presented for values of gene diversity (Additional file 4: Figure S2), PIC (Additional file 5: Figure S3) and minor allelic frequency (Additional file 6: Figure S4)**.** The overall mean value of polymorphic information content was 0.203 ranged from 0.01 to 0.375. Nei’s gene diversity score was varied from 0.01 to 0.5 with a mean value of 0.246 and the mean MAF of the genome was 0.175 ranged from 0.005 to 0.5. Chromosome 1A scored the highest PIC (0.229) and gene diversity (0.282) (Table 1). In contrast, the lowest PIC and genetic diversity score was observed on chromosome 7A (PIC = 0.181; gene diversity = 0.217). Chromosomes 2A, 2B, 3A, 3B, 7A and 7B showed slightly lower polymorphic information content than the average PIC values of the whole genome. On the other hand, homoeologous chromosome groups 1, 4, and 5 scored higher Nei’s genetic diversity than the average genome-wide value. The highest gene diversity, PIC and MAF were on homoeologous chromosome group five. Comparable mean values of genetic diversity, PIC and MAF were scored on A and B genomes.

**Table 1:** Mean values of diversity indices and minor allelic frequency distribution of SNP markers across chromosomes in 192 Ethiopian durum wheat accessions

Chromosome	Nei’s Gene diversity	Polymorphic information content (PIC)	Minor allelic frequency (MAF)
A-Genome			
1A	0.282	0.229	0.208
2A	0.221	0.185	0.150
3A	0.246	0.202	0.175
4A	0.248	0.204	0.176
5A	0.272	0.221	0.200
6A	0.265	0.215	0.194
7A	0.217	0.181	0.150
B-Genome			
1B	0.254	0.208	0.185
2B	0.229	0.190	0.158
3B	0.233	0.194	0.159
4B	0.279	0.226	0.209
5B	0.265	0.217	0.191
6B	0.246	0.203	0.172
7B	0.235	0.193	0.164
Homoeologous			
1	0.259	0.211	0.188
2	0.226	0.188	0.155
3	0.238	0.197	0.166
4	0.264	0.215	0.193
5	0.268	0.218	0.194
6	0.255	0.209	0.182
7	0.226	0.187	0.157
A-Genome	0.2448	0.2015	0.1744
B-Genome	0.2471	0.2035	0.1750
Whole Genome	0.2462	0.2027	0.1748

### Genetic stratification and principal component analysis

**Fig. 3:**
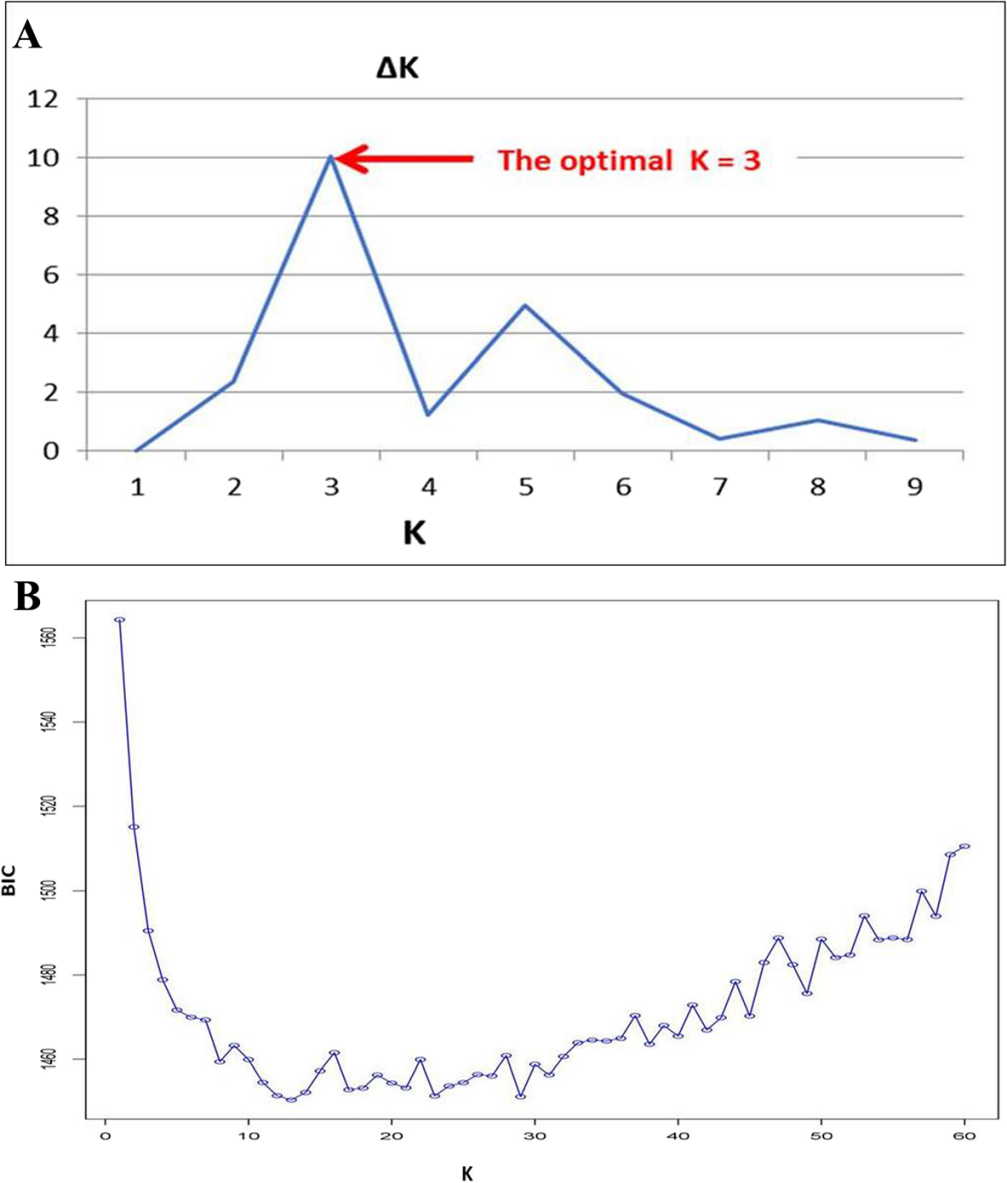
Inference of the optimal numbers of sub-populations (clusters) in Ethiopian durum wheat panel with the Bayesian clustering model in STRUCTURE (**A**) and by the discriminant analysis of principal components (DAPC) using adegenet package (**B**)

**Fig. 4:**
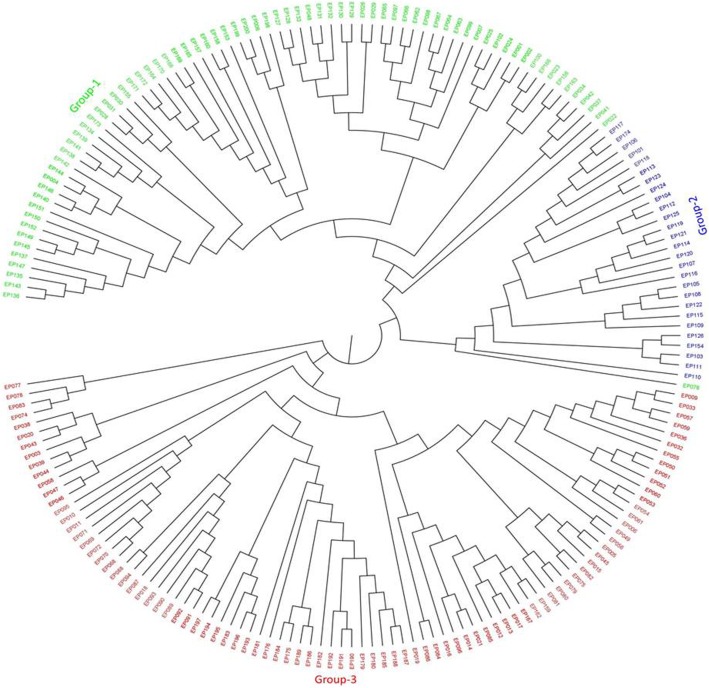
Neighbor-joining tree generated based on simple matching dissimilarity coefficients using SNP markers from 192 Ethiopian durum wheat accessions. Colors of accessions are clusters inferred from STRUCTURE-based analysis

The optimal sub-population of accessions was inferred through two approaches: The first method was the STRUCTURE-based clustering approach that was inferred based on the second order rate of change of the likelihood (∆K) (Table 3). The result indicated a clear peak at K = 3 signifying the optimal sub-populations in the panel (Fig. 3-a). The second approach was based on the discriminant analysis of principal components (DAPC) and the result couldn’t show a clear lowest Bayesian information criterion (BIC) on a specific K value above which BIC values decreased spontaneously with simultaneous increment making an elbow at the optimal K value (Fig. 3-b). However, in this case, it provided a clue in which somehow less than five clusters could be optimal. Hence, accessions were grouped into three clusters based on the STRUCTURE-inferred clustering result with 75, 27 and 90 accessions came together for sub-populations 1, 2 and 3, respectively (Additional file 1: Table S1). Landraces gathered on cluster-one and cluster-three while all improved varieties, except one variety (Selam) that was under cluster one, assembled on sub-population two. The neighbor-joining based clustering analysis (Fig. 4) also identified three clear clusters and except one accession all are grouped based on the STRUCTURE based stratification.

**Fig. 5:**
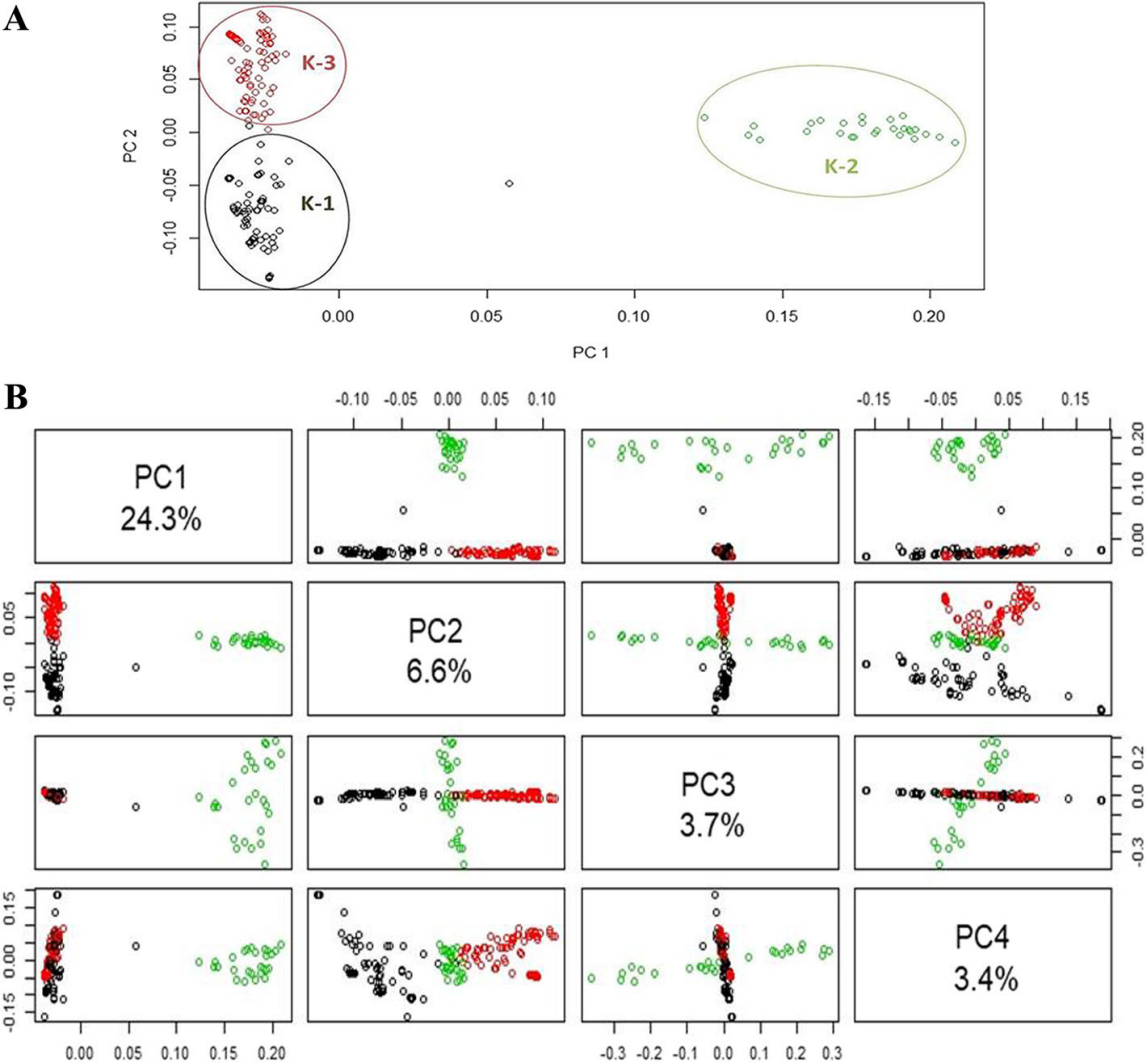
Principal component analysis (PCA) using 15,338 polymorphic SNP markers generated from 192 Ethiopian durum wheat accessions. (**A**) Varieties cluster (green, K-2) showing a clear separation from the other two landrace clusters (K1 & K2) using PC1 and PC2. (**B**) Clustering got disrupted when considering other PCs

Principal component analysis (PCA) was analyzed with all Polymorphic SNPs generated from the panel. The first, second and third principal components explained 24.29, 6.61 and 3.74% of the total variance, respectively. The smaller numbers of variance explained by the second and consecutive PCs indicated that only few PCs couldn’t encapsulate the existing genetic variance in Ethiopian durum wheat. The first PC (PC1) distantly clustered varieties from landraces and the second PC grouped the two landrace subgroups (Fig. 5-a). The first two PCs (PC1 and PC2) clearly clustered the three sub-populations**.** However, clustering gets distorted when additional principal components were considered (Fig. 5-b).

### Genetic divergence between landraces and improved varieties

The Bayesian model based stratification and PCA clustering methods grouped landraces and varieties in distinct places except in a single variety, Selam, which was clustered alongside landraces. The numbers of polymorphic SNPs were slightly higher in varieties than landraces. Varieties scored higher gene diversity (0.297), PIC (0.240) and the mean frequency of minor allele (0.218) than landraces (gene diversity = 0.213; PIC = 0.173 & MAF = 0.154) (Table 2).

**Table 2:** Mean of diversity indices and minor allelic frequency distribution of SNPs in landraces and varieties of Ethiopian durum wheat

Accession Type	Sample size	No. of polymorphic SNPs	Nei’s Gene diversity	Polymorphic information content (PIC)	Minor allelic frequency (MAF)
Landrace	167	13,466	0.213	0.173	0.154
Cultivar	25	13,725	0.297	0.240	0.218

**Table 3:** Inference of the optimal numbers of clusters existed in Ethiopian durum wheat panel using Delta K Statistics

K	L(K)	stdev	L’(K)	L”(K)	|L”(K)|	Delta K
1	−157,376	3.369	–	–	–	–
2	−138,930	1370.013	18,446.93	− 3224.75	3224.75	2.35
3	− 123,708	543.0884	15,222.19	− 5458.76	5458.76	10.05
4	−113,944	1149.967	9763.425	− 1419.46	1419.46	1.23
5	− 105,600	708.175	8343.965	− 3511.28	3511.28	4.96
6	−100,768	677.594	4832.685	− 1326.03	1326.025	1.96
7	−97,261	1353.421	3506.66	580.6019	580.6019	0.43
8	−93,173	1398.676	4087.262	− 1491.05	1491.054	1.07
9	−90,577	1708.376	2596.208	635.7119	635.7119	0.37
10	−87,345	2170.333	3231.92			

Where;

K = Subpopulations;

Ln (PD) = The log likelihood for each K;

L (K) = An average of 20 values of Ln P(D);

stdev = Standard deviation for 20 values of Ln P(D);

L’(K) = L(K) _n_ – L(K) _n-1_;

L”(K) = L′(K) n_+ 1_ − L′(K);

∆K = |L”(K)|/Stdev;

The gray color of the third line designates the optimal sub-populations of the panel based on the highest delta K value

**Table 4:** Analysis of molecular variance (AMOVA) for Ethiopian durum wheat accessions with and without grouping according to STRUCTURE clustering result

Source of variation	DF	Sum of squares	Variance components	Percentage of variation	Fixation indices	*P* value
Among populations	2	166,172.20	700.94 Va	52.41	FST = 0.52	Va and FST = 0.000
Among individuals Within populations	189	240,563.25	636.41 Vb	47.59	FIS = 1.00	Vb and FIS = 0.000
Total	191	406,735.45	1337.35

**Table 5:** Analysis of molecular variance) between landraces and varieties

Source of variation	DF	Sum of squares	Variance components	Percentage of variation	Fixation indices	P value
Among populations	1	108,435.72	1228.63 Va	61.02	FST = 0.61	Va and FST = 0.000
Among individuals Within populations	190	298,299.73	784.99 Vb	38.98	FIS = 1.00	Vb and FIS = 0.000
Total	191	406,735.45	2013.62			

However, the number of landraces included in the present study was higher by six folds than the number of varieties and that could affect the reliability of the PIC, genetic diversity and the minor allelic frequency scores reported in the current study.

### Genetic variation among clusters

The analysis of molecular variance (AMOVA) revealed the presence of higher genetic variance between STRUCTURE-inferred sub-populations (52.41%) than among individuals within clusters (47.59%) (Table 4).

Further analysis of molecular variance between the 25 varieties and 167 landrace accessions indicated higher genetic variation between the two groups (61.02%) than individuals within the group (38.98%) (Table 5).

### Genetic clustering via geographic origin

The current Ethiopian durum wheat germplasm comprises landrace accessions collected from major wheat-producing areas of the country (Additional file 3: Figure S1) including Bale, Gondar, Gojjam, Shewa, Tigray, and Wollo, and 12 Ethiopian durum wheat landraces currently cultivated in the USA.

The clustering analysis indicated that the SNPs data couldn’t group landraces clearly based on their geographical background and accessions were admixed into the different sub-groups irrespective to their geographic origin. For instance, eight landraces collected from northeastern Ethiopia (Wollo) were grouped in sub-population one while 25 landraces from the same origin clustered in sub-population three (Additional file 1: Table S1). Landraces collected from central Ethiopia (Akaki and Shewa) clustered in both sub-groups; 4 landraces in sub-population one while 19 landraces in sup-population three. However, from the total eight landraces collected in Bichena (a town in East Gojjam Zone, west-central Ethiopia), seven were grouped in sub-population three and the other one landrace altogether with four landraces collected from other parts of Gojjam were grouped in sub-population one. Landraces collected from Bale (Southeastern Ethiopia) grouped in both clusters (44 landraces in cluster 1 and 24 landraces in cluster 3). The two landraces collected from Tigray region (North Ethiopia) were clustered in sub-population one. However, a landrace collected from Gondar, adjacent to Tigray, was sub-grouped in cluster three. All twelve Ethiopian landraces that are now cultivated in the USA were gathered in sub-population three.

## Discussions

### Genetic diversity of Ethiopian durum wheat

Genetic diversity is imperative to provide a robust food security system capable of adapting to recurrent biotic and abiotic stresses. Genetic diversity analysis is a crucial step in noticing alleles that could be used as the source of novel traits with high yielding, resilient for biotic and/or abiotic stresses and yet delivers satisfied productivity or in meeting the end-user demands in plant breeding. Ethiopian durum wheat landraces have especially proven to show a relevant variation for various traits derived from their potential in adapting to changing environmental conditions [33]. Due to this, Ethiopian durum wheat germplasm has served as a center of focus for genetic studies and served as the source of novel QTLs, genes and gene complexes for many traits [9–14, 34].

Slightly higher number of SNPs (30,510) were reproduced in the present study from the 90 K wheat SNP array than previously reported by Mengistu et al. [12] on Ethiopian durum wheat (30,155 SNPs) and in Mediterranean durum wheat collections (21,069 SNPs). Genomes of A and B did not show a significant difference in diversity indices, indicating that they have followed similar evolutionary histories in Ethiopian durum wheat landraces and improved varieties [12].

The 90 K wheat SNP array was a platform made to capture the most reliable gene-associated SNP markers available in the wheat genome worldwide and could not enable to mine new loci. The less number of SNPs reproduced from the array in the current panel indicates the possibility of existence of novel alleles and further studies would be benefited from the employment of both hybridization and sequencing techniques to provide a thorough description of Ethiopian durum wheat genome.

Comparing with previous reports, higher genetic diversity indices were scored in Ethiopian durum wheat panel (Table 1) that strengthens the unresolved and ongoing argument of Ethiopia as the center of origin or domestication of durum wheat [3]. The result unveiled the presence of higher genetic diversity in Ethiopian durum wheat that could arise because of various causes including adaptation to wider agro-ecology [23], natural crossings due to cultivating mixed genotypes in a field and diverse farmers’ culture of agricultural practices [22, 35]. For instance, Ren et al. [36] reported mean polymorphic information content (0.18) and Nei’s gene diversity (0.22) from world-wide collected 150 durum wheat accessions genotyped with 1536 SNP markers. Kabbaj et al. [3] obtained a mean PIC value of 0.119 from 337 durum wheat accessions included landraces, varieties and elite lines collected from more than 30 countries genotyped with 35 K Affymetrix Axiom wheat breeders array. Eltaher et al. [37] reported slightly higher mean gene diversity (0.3) and PIC (0.23) in 250 winter wheat accessions genotyped with Genotyping-By-Sequencing (GBS) platform. However, unlike the present study, they only included SNP markers having less than 20% missing information and with minor allelic frequency (MAF) greater than 5%. As expected, higher PIC and genetic diversity scores were reported in studies using multi-allelic markers such as SSR, unlike SNPs, that could go beyond 0.5 values [38, 39].

### Genetic structure

Genetic stratification analysis based on the Bayesian clustering model of the second order rate of change of the likelihood [40] revealed the presence of three subpopulations. However, discriminant analysis of principal components based on the Bayesian information criterion (BIC) couldn’t show the smallest BIC on a specific K value above which the BIC values spontaneously decreased followed by simultaneous increment creating an elbow shape [41]. However, it provided a clue in which somehow less than five clusters could be optimal. Varieties showed a single distinct cluster and landraces distributed into two distinct clusters. Both principal component analysis (PCA) with the first two components and the neighbor joining clustering based on simple matching dissimilarity coefficient proved the former clustering result was optimal showing three clear clusters. In the current study, clustering was not based on their geographic origin where landrace accessions were originally collected in Ethiopia. Mengistu et al. [12] reported a similar result on a study conducted in 311 Ethiopian durum wheat accessions (287 landraces and 24 varieties) collected from major wheat producing areas of the country. This admixture could be due to the existence of historical and current exchange of seeds through informal seed system involving regional and countrywide farming communities [34]. Ren et al. [36] reported neither geographical nor ecological evidence was detected in grouping 150 durum wheat accessions with world-wide origin and noted that the possible reason could be the existence of gene flow via germplasm exchanges among different regions occurred frequently or that human transfer of genes in history made a very big admixture. Kabbaj et al. [3] found higher admixtures between 370 durum wheat accessions included landraces, varieties and elite lines collected from more than 30 countries including Ethiopia. However, they observed a very limited admixture between Ethiopian landraces with other collections originated world-wide and Ethiopian durum wheat landraces made a separate cluster and proved the presence of a unique morphology [10, 34] and represent a separate sub-species under the name *Triticum durum* subs. *Abyssinicum* or *T. aethiopicum* [22]. This phenomena placed Ethiopia as a secondary center of origin and diversity for durum wheat since the germplasm is distinct from the primary region of origin of durum wheat, the Fertile Crescent countries [3].

## Conclusions

In this study, 192 Ethiopian durum wheat accessions comprising 167 landraces and 25 improved varieties were assembled and genotyped with a high density 90 K wheat SNP array to analyze the existing genetic diversity and population structure within accessions. Clustering analysis showed a higher genetic admixture between landraces despite their geographic origin resulted from the existence of higher rate of historical seed exchange throughout the country. Diversity indices revealed the presence of higher genetic diversity in Ethiopian durum wheat accessions. Landraces adapted to wider agroecology and with the genetic capacity to tolerate various stresses could be used as a source of unique alleles in the enhancement of durum breeding through marker assisted selection or marker assisted backcrossing. Hence, sustainable conservation and utilization of Ethiopian durum wheat genetic resource is key for future breeding strategies in Ethiopia and worldwide.

## Methods

### Plant material

One hundred sixty seven Ethiopian durum landrace accessions collected from major wheat growing areas of the country and twenty five improved varieties released in different years and have been cultivated in Ethiopia were assembled for the present study. Improved varieties were released by Debre Zeit Agricultural Research Center (DZARC) and Sinana Agricultural Research Center (SARC), Ethiopia in different years (1994–2010). All landrace accessions and varieties are maintained by these two agricultural research centers as a single seed descent (SSD) progenies.

Landraces were originally collected from major wheat-producing areas of Ethiopia (Additional file 3: Figure S1) including Bale, Gondar, Gojjam, Shewa, Tigray, and Wollo, as well as twelve lines, which are originally from Ethiopia but currently cultivated in the USA. A detail of accessions is summarized in Additional file 1: Table S1.

### DNA extraction and SNP genotyping

A pooled tissue sample of twenty five one-week-old seedlings was taken for genomic DNA extraction for each accession. The DNA extraction was done with DNeasy 96 Plant Kit (Qiagen GmbH, Hilden, Germany).

SNP markers were generated using the Illumina iSelect® 90 K wheat SNP assay comprising 81,587 gene-associated SNPs [27]. Marker genotypes were called with the GenomeStudio v2011.1 software package (Illumina, San Diego, CA, USA) and calls showing residual heterozygosity were entered as missing values before exporting genotype data from the GenomeStudio. A high-density consensus map of tetraploid wheat generated by Maccaferri et al. [32] was used to identify chromosome positions of SNPs. The SNPs data used for diversity analysis is available in Additional file 2: Table S2.

### Genetic diversity analysis

Numbers and percent of polymorphic loci, polymorphism information content (PIC), Nei’s gene diversity and minor allelic frequency (MAF) were calculated using Power Marker v 3.25 [42]. PIC was estimated based on the probability of finding polymorphisms between any two random samples while Nei’s gene diversity defined as the probability of two randomly chosen alleles from the population is different. Principal component analysis (PCA) [43] for the genetic relationships among individuals was calculated using a package “SNPrelate” [44] in R studio [45]. Neighbor-Joining tree based on simple matching dissimilarity coefficient was constructed using DARwin var. 6.0.14 [46] and the resulting trees were displayed using FigTree var. 1.4.3 [47]. A software package Arlequin v.3.5.2.2 [48] was used to assess the molecular variance (AMOVA) between clusters based on STRUCTURE-inferred subpopulations and between landraces and varieties.

### Genetic structure analysis

Two approaches were implemented to infer the optimal clusters/subpopulations existed in 192 Ethiopian durum wheat accessions. First, a Bayesian model-based clustering approach was used to estimate the optimal subpopulations and the membership probability of each genotype to the subpopulations using STRUCTURE v.2.3 [49]. To infer the optimal clusters, an ad hoc quantity (∆K) approach was applied that was calculated based on the second order rate of change of the likelihood [40]. For this analysis, 10 sub-populations with 20 independent iterations for each sub-population was done under the admixture model of population structure with correlated allele frequencies and 50,000 lengths burn-in period and 100,000 Markov Chain Monte Carlo (MCMC) replications after burn-in was applied for each iteration.

The second approach was based on the discriminant analysis of principal components (DAPC) implemented using a package “adegenet” [41] in R studio. In this method, the optimal clustering solution corresponded to the lowest Bayesian Information Criterion (BIC) and the number of clusters determined as the value of K above which BIC values decreased with simultaneous increment making an elbow at the optimal cluster [41].

## Supplementary information

**Additional file 1 **: **Table S1.** Accession names and types, cultivated areas, seed sources and population structure of 192 Ethiopian durum wheat accessions used for the current study.

**Additional file 2 **: **Table S2.** List of polymorphic SNPs, their alleles and chromosomes used for genetic diversity analysis in Ethiopian durum wheat accessions.

**Additional file 3 **: **Figure S1.** Map of Ethiopia showing the major wheat producing areas where 167 landraces were originally collected (Source: Atlas of the Ethiopian rural Economy (2006); http://www.ifpri.org/node/3763).

**Additional file 4 **: **Figure S2.** Frequency distributions for Nie’s gene diversity score of polymorphic SNPs across chromosomes in Ethiopian durum wheat accessions.

**Additional file 5 **: **Figure S3.** Frequency distributions for polymorphic information content (PIC) values of polymorphic SNPs across chromosomes.

**Additional file 6 **: **Figure S4.** Frequency distributions for minor allele frequency (MAF) values of polymorphic SNPs across chromosomes.

## Data Availability

The data sets supporting the results of this article are included in this manuscript and its additional information files.

## Abbreviations

AMOVA: Analysis of molecular variance
BIC: Bayesian information content
DAPC: Discriminant analysis of principal components
DZARC: Debre Zeit agricultural research center
EBI: Ethiopian biodiversity institute
MAF: Minor allele frequency
MCMC: Markov chain Monte Carlo
PCA: Principal component analysis
PIC: Polymorphic information content
SARC: Sinana agricultural research center
SNP: Single nucleotide polymorphism
