# Supplementary figures and images for: Genetic diversity and population structure analysis based on the high density SNP markers in Ethiopian durum wheat (Triticum turgidum ssp. durum)
Source: BMC Genet. 2020 Feb 12;21:18. doi: 10.1186/s12863-020-0825-x (PMC7017545; doi:10.1186/s12863-020-0825-x)

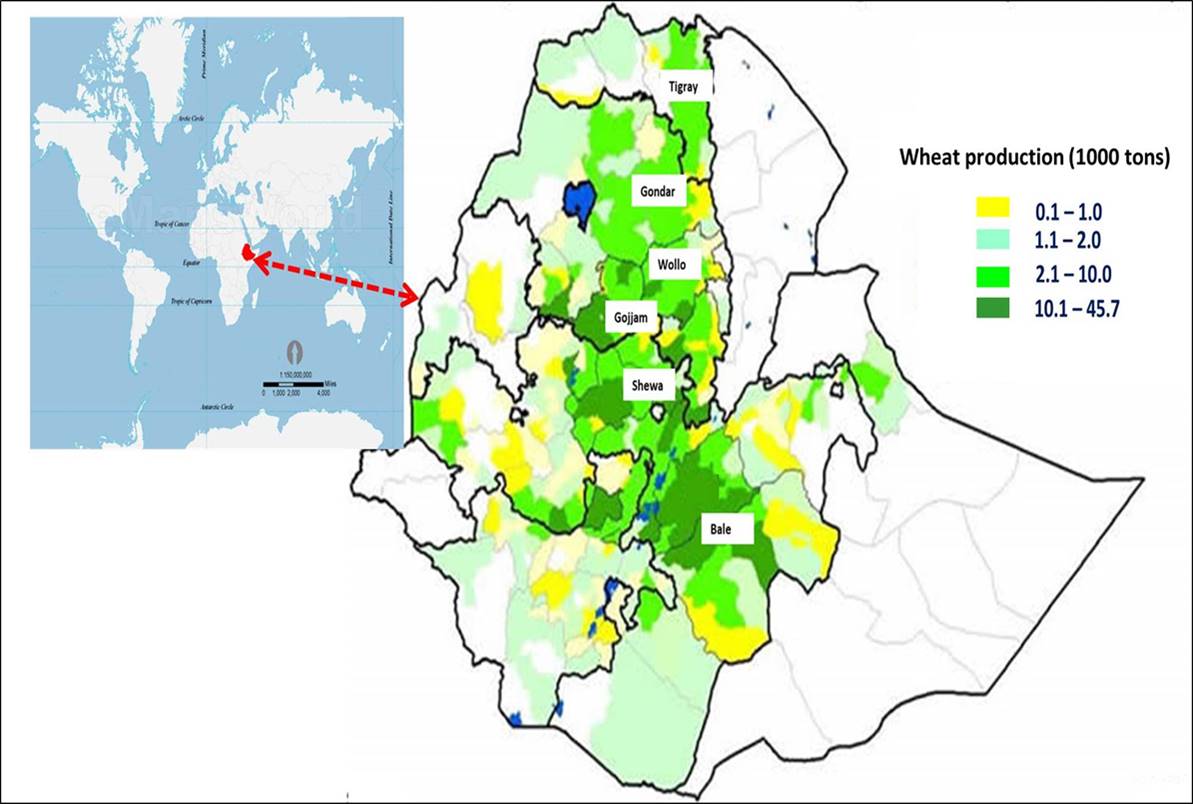

Supplement: Supplementary file 3 — Additional file 3 : Figure S1. Map of Ethiopia showing the major wheat producing areas where 167 landraces were originally collected (Source: Atlas of the Ethiopian rural Economy (2006); http://www.ifpri.org/node/3763). [file 12863_2020_825_MOESM3_ESM.jpg]

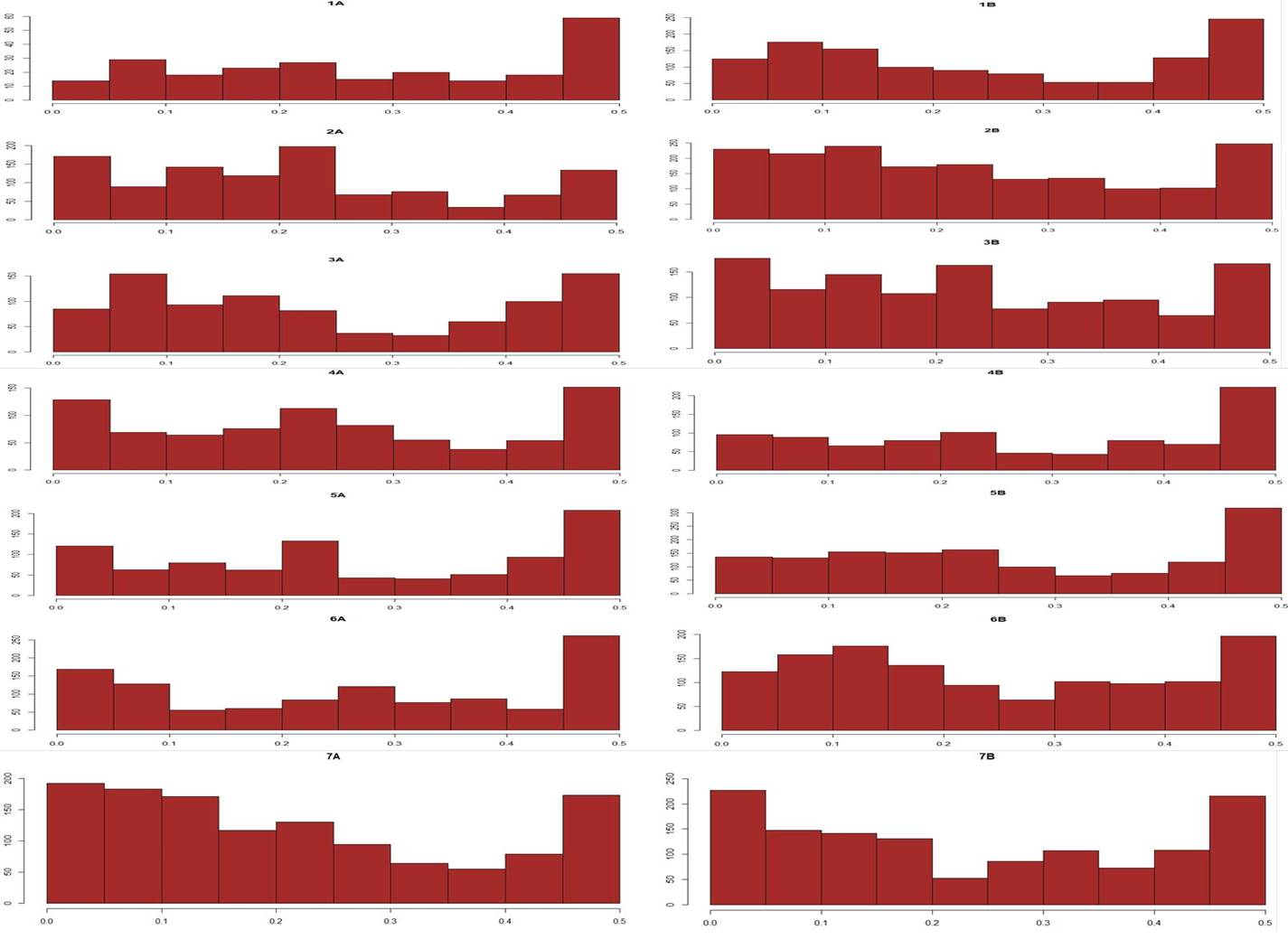

Supplement: Supplementary file 4 — Additional file 4 : Figure S2. Frequency distributions for Nie’s gene diversity score of polymorphic SNPs across chromosomes in Ethiopian durum wheat accessions. [file 12863_2020_825_MOESM4_ESM.jpg]

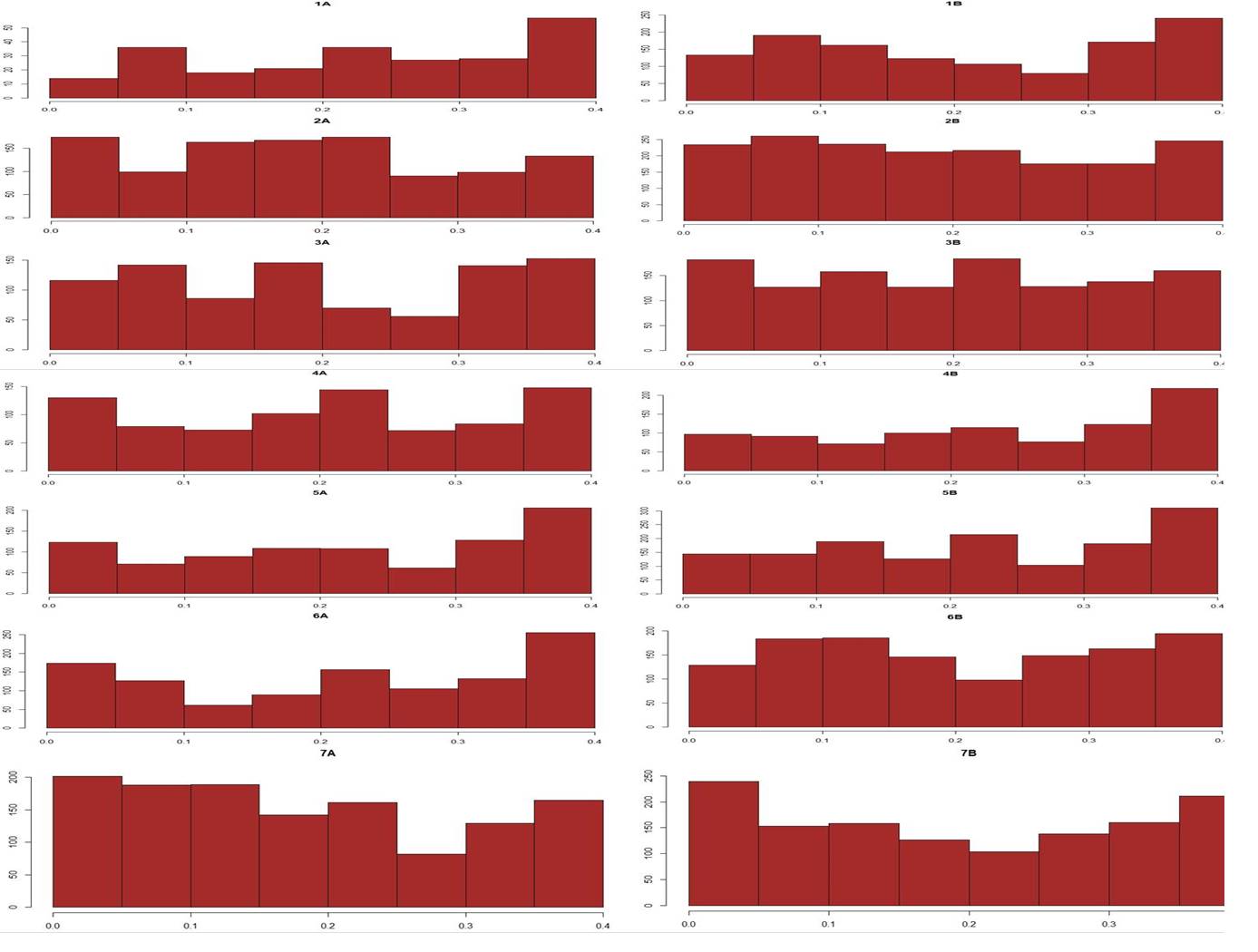

Supplement: Supplementary file 5 — Additional file 5 : Figure S3. Frequency distributions for polymorphic information content (PIC) values of polymorphic SNPs across chromosomes. [file 12863_2020_825_MOESM5_ESM.jpg]

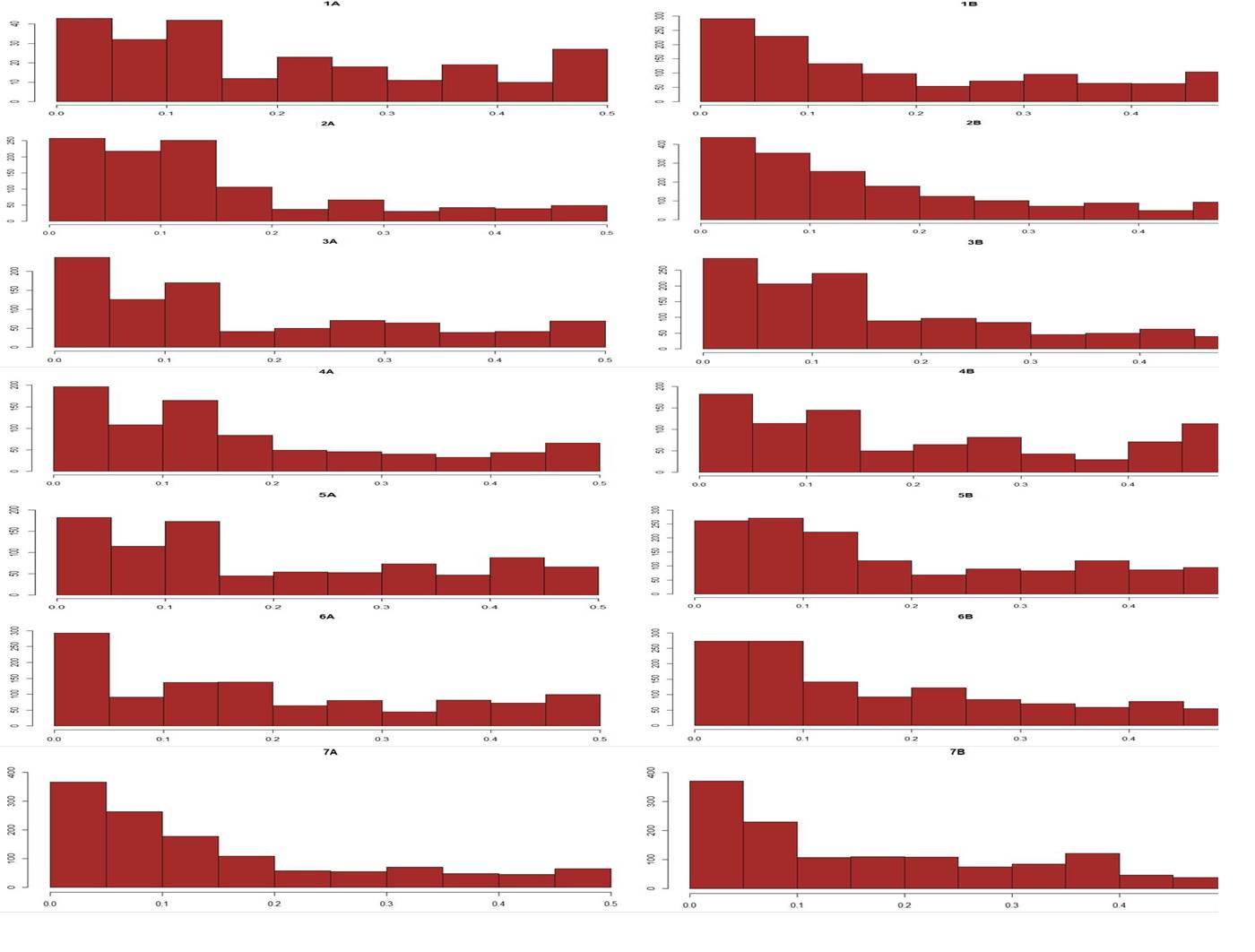

Supplement: Supplementary file 6 — Additional file 6 : Figure S4. Frequency distributions for minor allele frequency (MAF) values of polymorphic SNPs across chromosomes. [file 12863_2020_825_MOESM6_ESM.jpg]
